# Single-Cell RNA-seq reveals transcriptomic modulation of Alzheimer’s disease by activated protein C

**DOI:** 10.18632/aging.205624

**Published:** 2024-02-21

**Authors:** Mohammad Kasim Fatmi, Hao Wang, Lily Slotabec, Changhong Wen, Blaise Seale, Bi Zhao, Ji Li

**Affiliations:** 1 Department of Surgery, Morsani College of Medicine, University of South Florida, Tampa, FL 33612, USA; 2 Department of Physiology and Biophysics, University of Mississippi Medical Center, Jackson, MS 39216, USA; 3 Genomics Program, College of Public Health, University of South Florida, Tampa, FL 33612, USA; 4 G.V. (Sonny) Montgomery VA Medical Center, Jackson, MS 39216, USA

**Keywords:** APC, Alzheimer’s disease, inflammation

## Abstract

Single-Cell RNA sequencing reveals changes in cell population in Alzheimer’s disease (AD) model 5xFAD (5x Familial AD mutation) versus wild type (WT) mice. The returned sequencing data was processed through the 10x Genomics *CellRanger* platform to perform alignment and form corresponding matrix to perform bioinformatic analysis. Alterations in glial cells occurred in 5xFAD versus WT, especially increases in microglia proliferation were profound in 5xFAD. Differential expression testing of glial cells in 5xFAD versus WT revealed gene regulation. Globally, the critical genes implicated in AD progression are upregulated such as *Apoe*, *Ctsb*, *Trem2*, and *Tyrobp*. Using this differential expression data, GO term enrichment was completed to observe possible biological processes impacted by AD progression. Utilizing anti-inflammatory and cyto-protective recombinant Activated Protein C (APC), we uncover inflammatory processes to be downregulated by APC treatment in addition to recuperation of nervous system processes. Moreover, animal studies demonstrated that administration of recombinant APC significantly attenuated Aβ burden and improved cognitive function of 5xFAD mice. The downregulation of highly expressed AD biomarkers in 5xFAD could provide insight into the mechanisms by which APC administration benefits AD.

## INTRODUCTION

Alzheimer’s Disease (AD) causes neuroinflammation characterized by the progressive degradation of neurological functions that eventually result in memory loss and confusion [1–3]. AD pathology has widely pointed to amyloid-beta (Aβ) production in the brain to be a primary source for neurodegeneration and has been the key therapeutic target for AD treatment [4]. Over 6.5 million Americans currently fall victim to Alzheimer’s dementia with the number of victims projected to grow year over year as AD remains the 6th leading cause of death in the United States [5, 6].

Activated Protein C (APC) is a plasma zymogen that has shown promising cytoprotection, anti-inflammation, and anti-apoptotic properties [7]. APC is neuroprotective, and the anti-inflammatory property particularly is attributed to neuroprotection in translational studies [8–10]. APC treatment in our preceding studies have been found to positively treat ischemia/reperfusion (I/R) injury and cardiac dysfunction in myocardial infarction mice [11]. We aim to modulate AD and reverse many of its symptoms using exogenous APC treatment. Preeminently, APC treatment has been found to act on Aβ production by regulation of β-Secretase in 5xFAD transgenic mice, possibly providing rationale towards the transcriptional regulation observed in this study [12]. In current amyotrophic lateral sclerosis (ALS) and stroke human trials, APC treatment resulted in positive hemorrhage reduction paving the way for future studies of APC for AD in humans [13, 14]. We believe our study utilizing Single-Cell RNA sequencing (scRNA seq) will provide additional rationale towards the effects on APC treatment observed in previous studies on 5xFAD AD mice.

In our investigation, we utilized transgenic mice that contain expression for five major amyloid pathologies that allow for rapid progression of AD and Aβ deposition known as 5xFAD mice [15, 16]. Previous research in the field has identified crucial pathological differences between wild type (WT) and 5xFAD mice that provide insight towards genetic variables correlated with AD [17]. Specifically, 5XFAD mice overexpress the K670N/M671L (Swedish), I716V (Florida), and V717I (London) mutations in human APP (695), as well as M146L and L286V mutations in human PS1. Our goal is to employ an innovative approach to not only uncover differential transcriptional expression between 5xFAD and WT mice but also identify specific neurological cell-types impacted by the onset of AD. The method of choice to complete this study was scRNA seq which will allow us to examine differential expression on a cell-type level versus previous studies that employed inferior methods observing global transcriptional variations [18].

Evolving our previous approach to scRNA seq analysis, the essential integration feature of *Seurat v4* once more allowed us to do a comprehensive study between samples [19, 20]. Using canonical marker genes to annotate cells into six neurological cell-types for comparison between samples: Astrocytes, Endothelial cells, Microglia, Neuron, Oligodendrocyte, and Oligodendrocyte Precursor Cells (OPC). While many preceding studies have investigated AD progression transcriptionally [21–23]. Our sc-RNA seq approach provides a new unique perspective on the treatment of AD using exogenous APC. Our study not only observes the transcriptome of 5xFAD mice compared to WT on six main neurological cell-types but measures the impact of APC treatment on the same cell-types. The changes in cell population and transcriptome regulation in each cell-type allows for a greater investigation into phenotypic and physiological alterations occurring during the onset of AD and a five-month APC treatment.

## RESULTS

### Integration and annotation

**Figure 1:**
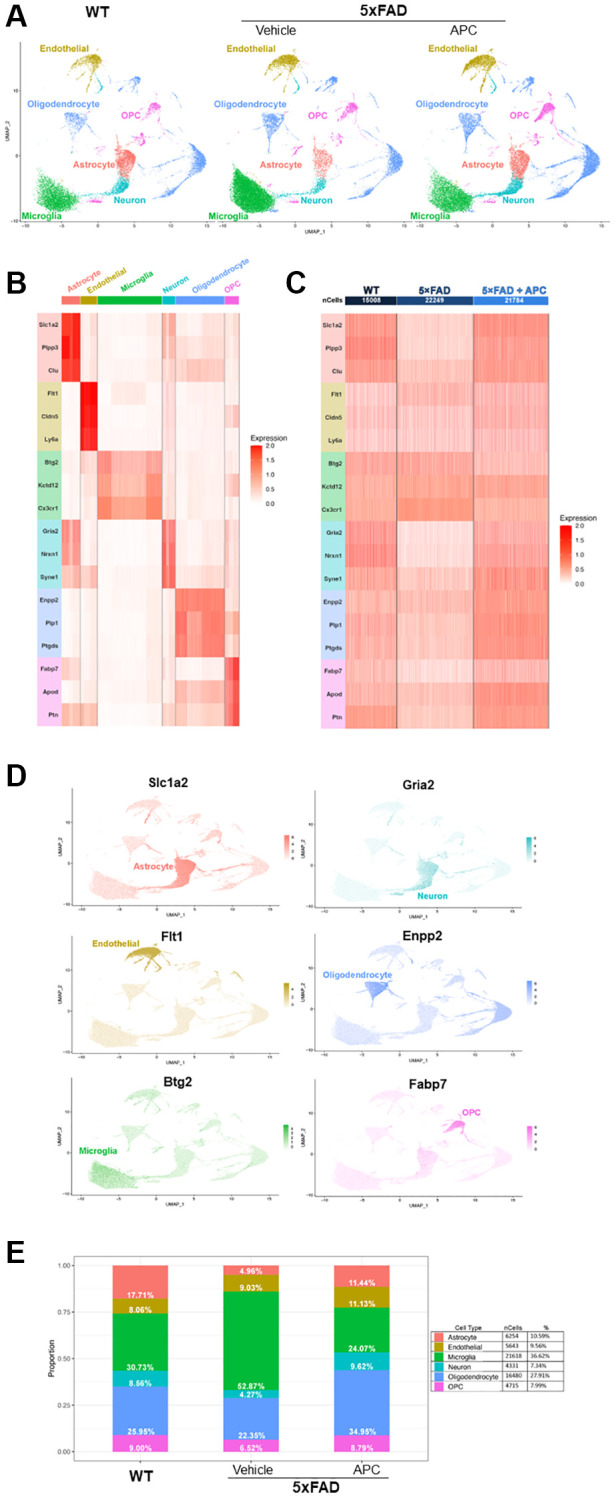
(**A**) Uniform Manifold Approximation and Projection (UMAP) dimensional plot of the primary integrated dataset split by sample; list of unsupervised clusters colored by cell-type annotated. Wildtype (left), 5xFAD (center), 5xFAD + APC (right). (**B**) Heatmap of top two conserved marker gene expression in each cluster used for cell-type annotation. Gene color corresponds to cell-type annotation. (**C**) Heatmap of top two conserved marker gene expression in each dataset used for cell-type annotation. Gene color corresponds to cell-type annotation. Number of cells shown per sample. (**D**) Feature plot of top marker genes, coloring cells by expression of the top marker gene for each cell-type annotated. (**E**) Proportion and percentage of each cell-type observed in each individual sample as well as the overall integrated dataset.

Generally following the Satija Lab integration vignette [19], integrated datasets were made utilizing four sample conditions from wild type C57BL/6 mice w/o the administrator of APC (WT, WT+APC), and 5xFAD C57BL/6 mice with the administrator of APC (5xFAD, 5xFAD+APC). These integrated datasets allowed for observing transcriptional differences between 5xFAD + APC, 5xFAD, and WT mice and 20 unsupervised clusters were generated (Figure 1A). Additionally, a control dataset was generated to compare WT + APC versus WT (Supplementary Figure 1A). The *Seurat* integration workflow generates a combined dataset that achieves the maximum overlap of cells from both samples to annotate cell-types [19]. This ensured that each cluster annotated contained cells from both samples within the integrated dataset as much as possible.

The top three highly expressed conserved genes in each unsupervised cluster were used for cell-type annotation (Figure 1B, 1C). Of the 20 unsupervised clusters, only 17 expressed at least three canonical marker genes and were retained for further downstream analysis. Astrocyte cell populations were annotated by high expression of *Slc1a2*, *Plpp3*, and *Clu*. Endothelial cell populations were annotated with remarkable expressions of *Flt1*, *Cldn5*, and *Ly6a*. Microglial cell populations were chosen based on expression of markers *Btg2*, *Kctd12*, and *Cx3cr1*. Neuron cell populations were annotated with expression of *Gria2*, *Nrxn1*, and *Syne1*, though overlap with astrocyte cells were noted. Oligodendrocytes were annotated by *Enpp2*, *Plp*, and *Ptgds* markers and OPCs were annotated on expression of *Fabp7*, *Apod*, and *Ptn*. The top marker gene can be visually observed in the dimensional projection (Figure 1D).

### Cell population

After cell-type annotation, the proportion of cell-types that form each sample can be observed and provide insight on critical alterations in cell population between samples (Figure 1E). Notably, the proportion of microglia in 5xFAD mice is significantly greater than their WT counterparts, making up over half the cells recovered. Decreases in astrocyte and neuron populations are also observed in 5xFAD mice, both populations reducing to ~4%. After introduction of exogenous APC, a rebound of impacted cell-types can be observed. Astrocyte and neuron cell populations return to near-WT levels as well as an incredible decrease in microglia cell population in APC-treated 5xFAD mice. The astrocyte population returns to roughly 11% of cells and neurons reached their greatest proportion after APC treatment nearly 10%. Microglial cells are cut in half, decreasing from ~53% in 5xFAD mice to 24% in APC treated mice. Notably, oligodendrocytes are also at their largest proportion following APC treatment at ~35%.

Globally, some 59,000 cells are contained in the main integrated dataset, ~15,000 from WT, ~22,000 from 5xFAD, and ~22,000 from 5xFAD with APC treatment (Figure 1C). Of these cells, 6,254 astrocyte cells were identified, making up just over 10% of the overall dataset. 5,643 endothelial cells were identified at just under 10% globally. Most of the dataset is made up of microglial cells with 21,618 cells identified and making up ~37% of total cells. The least number of cells identified were neurons with 4,331 cells that form ~7% overall. Oligodendrocytes are the second most abundant cell-type with 16,480 cells identified making up ~28% overall. Finally, OPCs make up 8% of the total cells in the dataset with 4,715 cells identified (Figure 1E). In the control dataset, APC-treated WT mice do not exhibit drastic changes in cell populations (Supplementary Figure 1B). Astrocyte, endothelial cell, microglia, neuron, oligodendrocyte, and OPC populations remain largely unaffected by exogenous APC nullifying major adverse effects in the baseline. We observed 19 DEGs (Log_2_FC > ± 0.3, adj. *p*-value < 0.05) globally between WT with APC treatment and WT (Supplementary Figure 1C).

### Differential expression

**Figure 2:**
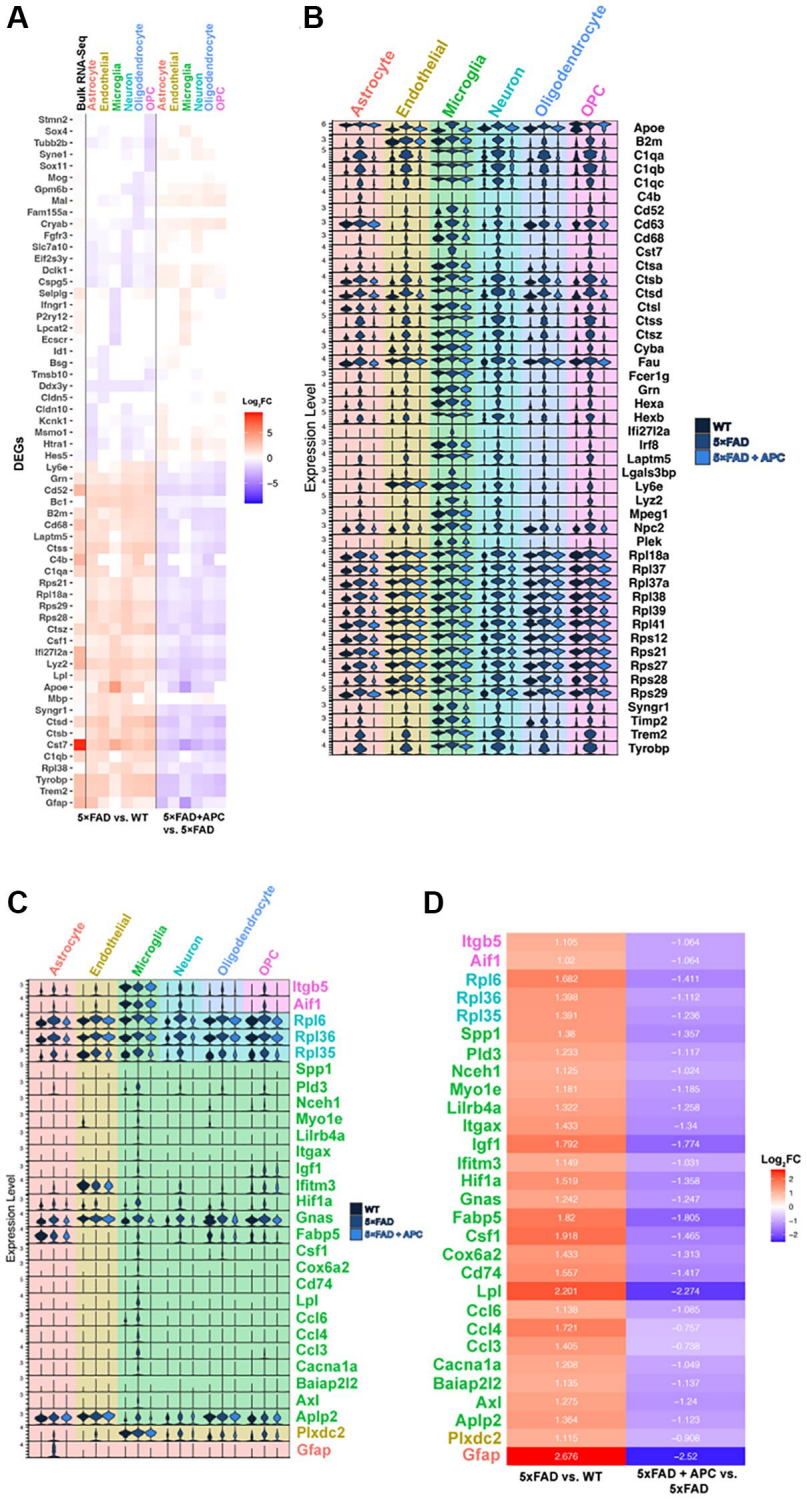
(**A**) Top 5 most significant DEGs (*p*-value = 0) per cell type in 5xFAD versus WT (left) and corresponding expression with APC-treatment (right). Bulk RNA-seq shown in column furthest left (6-month 5xFAD vs. WT). (**B**) Stacked violin plot of the top global DEGs (*p*-value < 0.05) found in multiple cell-types. (**C**) Stacked violin plot of unique DEGS highly differentiated within a particular cell-type. (**D**) Corresponding heatmap of unique DEGs highly differentiated in a particular cell-type (Figure 2C) plotted with Log2FC value in that cell-type. 5xFAD versus WT (left), 5xFAD + APC versus 5xFAD (right).

Once stable cell-types were established, a wide variety of differentially expressed genes (DEGs) in all cell-types were found. The top five DEGs per cell-type were identified and cross-referenced with bulk-RNA seq (Figure 2A). We obtained the bulk RNA sequencing data from GSE140286. The log_2_FC for each gene were calculated by comparison of 6-month 5xFAD to 6-month WT using *Limma* Package. We mapped genes in GSE140286 with the top DEGs list from scRNA sequencing. Comparing the DEGs between bulk and single-cell sequencing reveals that genes we observed to be upregulated in single cell-types in 5xFAD mice have been found to be generally upregulated in bulk sequencing (Figure 2A). We did not observe the DEGs that were downregulated in 5xFAD mice in our dataset to broadly match in the bulk sequencing data.

Consequently, we focused on genes that are greater expressed in 5xFAD compared to WT and later strongly downregulated by APC treatment (Supplementary Figure 2A, 2B). Generally, we highlighted exceptionally expressed genes meeting a threshold of Log_2_FC values ~1 resulting to 77 total DEGs across all cell-types. Many DEGs are globally upregulated in all cell-types in 5xFAD compared to WT. We identified 46 global DEGs that are all remarkably expressed in all 5xFAD cell-types and downregulated with APC treatment (Figure 2B). Many of these globally expressed genes are associated with the transgenic nature of 5xFAD and the onset of AD, their roles in each cell-type are of particular interest. The implications of these global DEGs may be more impactful in a specific cell-type versus another such as *Ctsb* in microglia, further investigation is needed to verify the physiological impact of specific genes in AD treatment.

Within astrocytes, *Gfap* particularly is abundantly found in the astrocyte cell population and profusely upregulated in 5xFAD (Figure 2C). Compared to WT, AD astrocytes have a 2.68 log_2_FC increase in *Gfap* expression; after APC treatment, *Gfap* became significantly downregulated with a 2.52 log_2_FC decrease in expression (Figure 2D). Many global DEGs including *B2m*, *C1qa*, *Ctsb*, *Trem2*, and *Tyrobp* are all impressively down regulated in 5xFAD + APC (Figure 2B). We find the DEGs found in endothelial cells appear to be non-exclusive. *Plxdc2* are broadly expressed in other cell-types; however, are only strongly differentially expressed in endothelial cells (Figure 2C). *Plxdc2* receives a 1.11 log_2_FC expression increase followed by a 0.91 log_2_FC decrease in expression with APC (Figure 2D). Like found in astrocytes, *Apoe*, *C1qa*, *Ctsb*, *Trem2*, *Tyrobp*, etc. were found to be especially upregulated in endothelial cells of 5xFAD mice (Figure 2B). These genes are not necessarily specific to endothelial cells; however, the effects of these DEGs on endothelial cells may provide insight on neurodegeneration. These DEGs get significantly downregulated after APC treatment in endothelial cells. The importance of any single gene in endothelial cells specifically is not well understood.

Microglial cells are widely associated with the progression and pathology of AD. Many DEGs that are globally upregulated are the most implicated in microglia regarding AD. These include genes: *Apoe*, *Cst7*, *Cd63*, *Cd68*, cathepsins (*Ctsa*, *Ctsb*, *Ctsd*, ect.), *Irf8*, *Trem2*, and *Tyrobp*. Though most of the DEGs are modulated by APC treatment, interestingly, *C1qa*, *C1qb*, *C1qc*, *Hexa/b*, *Laptm5* and *Ly6e* do not seem to alter in expression level extensively in microglia (Figure 2B). The microglial-exclusive DEGs most significantly modulated by APC were *Aplp2*, *Axl*, *Baiap2l2*, *Cacna1a*, *Ccl3*, *Ccl4*, *Ccl6*, *Lpl*, *Cd74*, *Cox6a2*, *Csf1*, *Fabp5*, *Gnas*, *Hif1a*, *Ifitm3*, *Igf1*, *Itgax*, *Lilrb4a*, *Myo1e*, *Nceh1*, *Pld3*, and *Spp1* (Figure 2C). The downregulation of the cathepsin family of genes by APC treatment is distinctly remarkable (Figure 2C).

In neurons, like endothelial cells, the DEGs found are expressed in various cell-types though firmly differentially expressed in neurons (Figure 2C). Robust DEGs in 5xFAD neurons with a greater than ~1.4 log_2_FC increased expression include *Rpl35*, *Rpl36*, and *Rpl6* (Figure 2D). It was found that APC treatment downregulated all the mentioned genes in neurons with greater than a 1 log_2_FC decrease in expression (Figure 2D). Oligodendrocyte DEGs in 5xFAD also widely consist of global upregulated genes (Figure 2B). The vast majority of global DEGs in oligodendrocytes were also found to be downregulated by APC treatment (Figure 2B). This may implicate the roles of these DEGs in the large oligodendrocyte proliferation found in APC treated 5xFAD mice (Figure 1D). Global DEGs were also found in OPCs including *B2m*, *C1qa*, *Cst7*, cathepsins, *Syngr1*, *Trem2*, and *Tyrobp* (Figure 2B). We found *Aif1* and *Itgb5* to be remarkably differentially expressed in OPCs of 5xFAD mice (Figure 2C) Measuring a ~1 log_2_FC increased expression in 5xFAD mice compared to WT and a corresponding ~1 log_2_FC decreased expression with APC treatment (Figure 2D). Accordingly, wide downregulation of global DEGs were also observed in OPCs of APC treated mice akin to oligodendrocytes (Figure 2B).

### Microglial alterations

**Figure 3:**
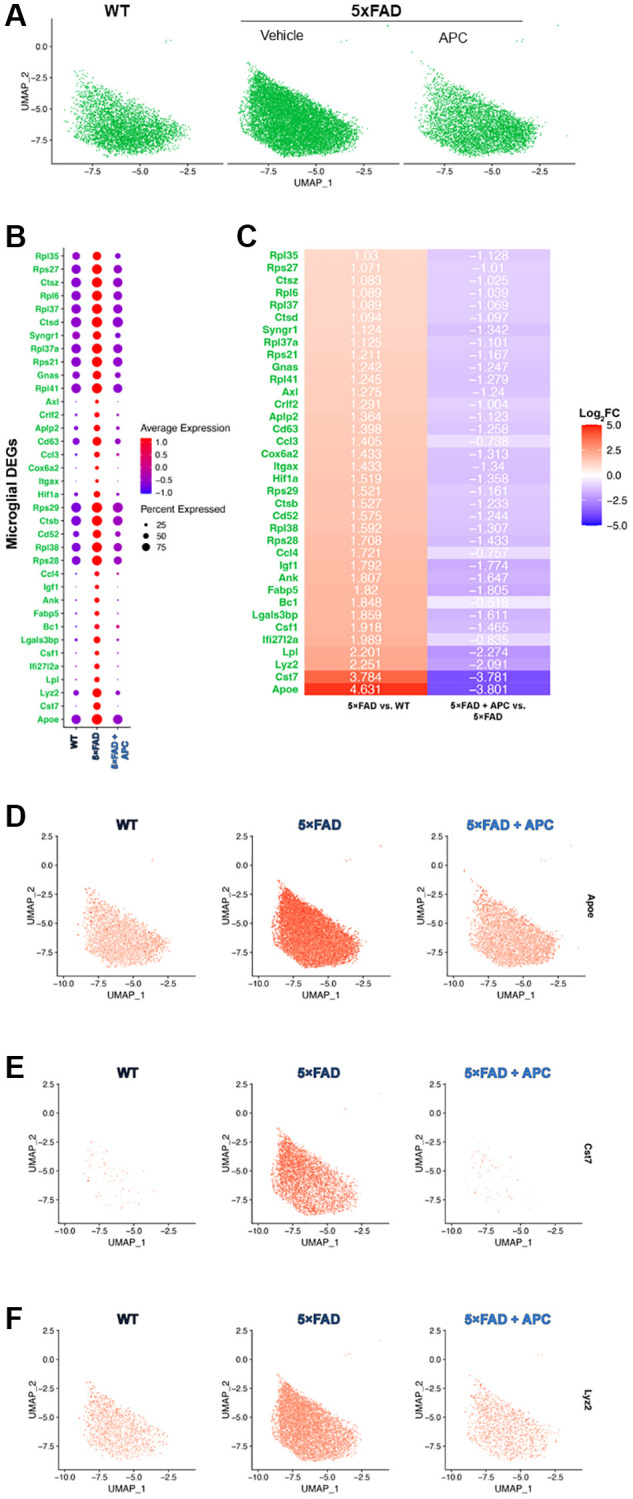
(**A**) UMAP dimensional plot of the microglial cell subset from the primary integrated dataset (Figure 1A) split by original sample, WT (left), 5xFAD (middle), 5xFAD + APC (right). (**B**) Dot plot of the top DEGs impacted by APC-treatment (Log2FC > 1, *p*-value = 0) in the microglial subset. (**C**) Corresponding heatmap of the top DEGs most impacted by APC-treatment in the microglial subset. Plotted with Log2FC value, 5xFAD versus WT (left), 5xFAD + APC versus 5xFAD (right). (**D**–**F**) Split feature plot of the top 3 microglial DEGs: Apoe, Cst7, and Lyz2 expression in each sample respectively.

Sub-setting the microglial population allowed for further transcriptome analysis of the ~22,000 cells identified in the original dataset (Figure 3A). We discovered ~900 significant DEGs (adj. *p*-value < 0.05) between the samples (Supplementary Figure 2C). As with our comprehensive study, we targeted DEGs that were strongly impacted by APC-treatment. We identified 36 microglial DEGs with a Log_2_FC > 1 and an adjusted *p*-value of 0 (Figure 3B, 3C). A large portion of these DEGs were previously identified in the comprehensive differential expression testing (Figure 2B, 2C) including *Aplp2*, *Ccl4*, *Fabp5*.

We measure that the top genes (Log_2_FC >2) that were expressed in 5xFAD microglia were *Apoe*, *Cst7*, *Lyz2*, and *Lpl* (Figure 3C–3F). These were all strongly downregulated with APC treated microglia (Figure 3C) as observed in multiple cell-types during the comprehensive results (Figure 2B). Many of the broadly regulated DEGs found previously (Figure 2B) remain largely implicated in the microglial population. Major lysosomal factors *Ctsb*, *Ctsd*, and *Ctsz* were found to be significantly differentially expressed in microglia (adj. *p*-value = 0) of 5xFAD and APC treated mice. Our subset further uniquely found additional DEGs that were not found during the differential expression testing of all cell-types. *Ank*, *Crlf2*, *Rpl35*, and *Rpl38* were differentially expressed in the microglial cell population but were not found during previous testing comprehensively.

### GO term enrichment

**Figure 4:**
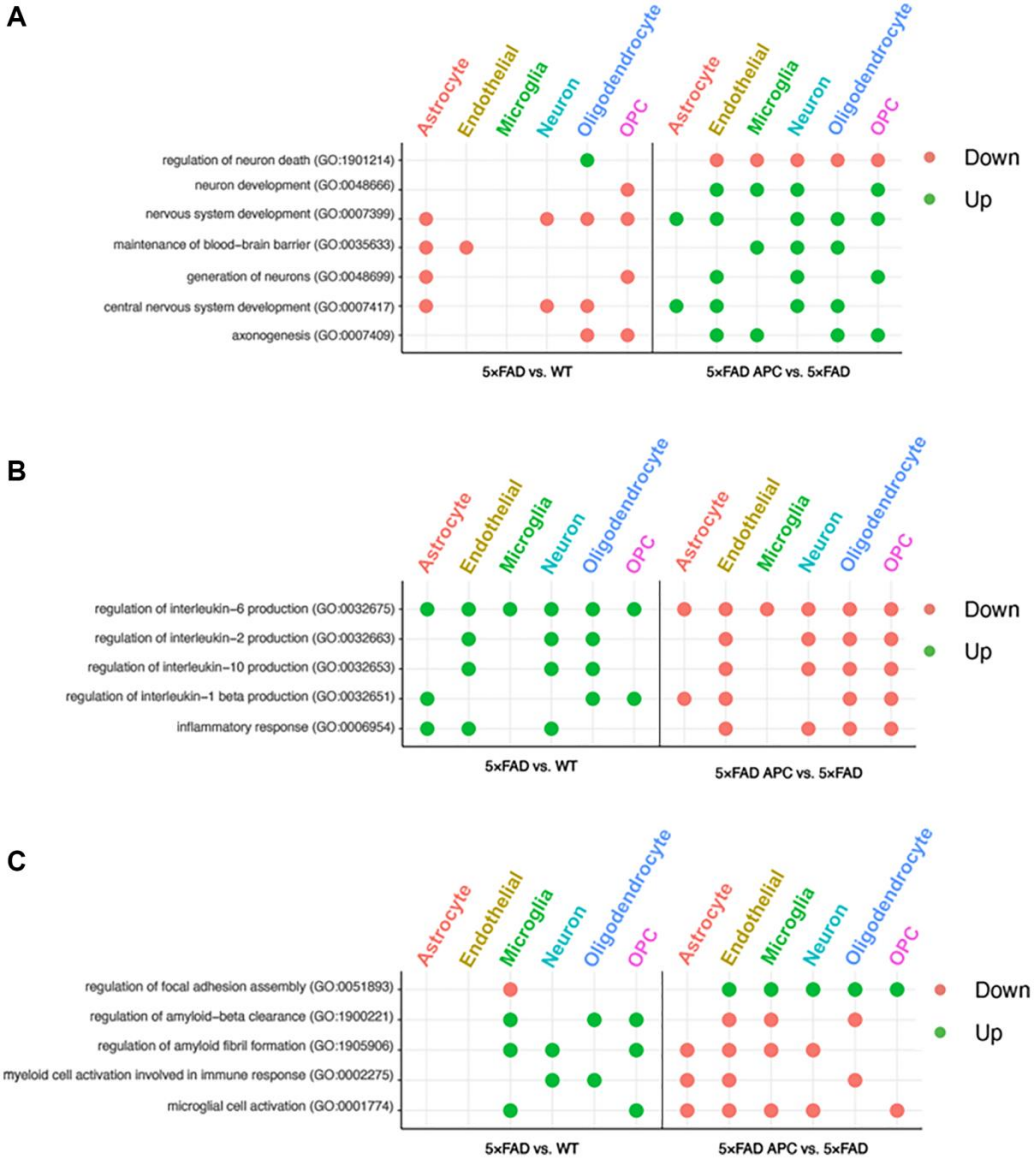
(**A**) Enriched GO terms in each cell-type from 5xFAD versus WT (left) and 5xFAD + APC versus 5xFAD (right) pertaining to neural development and nervous system. (**B**) Enriched GO terms in each cell-type from 5xFAD versus WT (left) and 5xFAD + APC versus 5xFAD (right) pertaining to immune response and inflammation. (**C**) Miscellaneous enriched GO terms in each cell-type from 5xFAD versus WT (left) and 5xFAD + APC versus 5xFAD (right).

The data generated by differential expression testing allowed for GO term enrichment. This enrichment data provides insight towards the roles of each cell-type in AD progression as well as during APC treatment. Universally, every enriched GO term for 5xFAD cells as a reciprocal in APC treatment (Figure 4A–4C). Our enrichment data shows 5xFAD oligodendrocyte and OPCs downregulate axonogenesis followed by counter upregulation in APC treated mice. Endothelial cells and microglia upregulated axonogenesis in addition to the oligodendrocyte and OPCs in APC treatment (Figure 4A). Central nervous system and nervous system development is downregulated in astrocyte, neuron, and oligodendrocytes in 5xFAD mice (Figure 4A). Corresponding upregulated nervous system development in APC treated mice can be found in every cell-type except for microglia. Downregulation of neuron generation is found in astrocyte and OPCs in 5xFAD mice and is regulated in endothelial cells, neurons, and OPCs of APC treated mice (Figure 4A). 5xFAD astrocyte and endothelial cells were found to downregulate blood-brain barrier (BBB) maintenance and compelling upregulation of BBB maintenance was found in the microglia, neuron, and oligodendrocytes of APC treated mice (Figure 4A). AD mice exhibit considerable neuron death (Figure 4A). Neuron development is downregulated in OPCs, and neuron death is upregulated in oligodendrocytes in 5xFAD mice. Endothelial cells, microglia, neurons, oligodendrocytes, and OPCs all are implicated in the rebound of neurons of APC treated mice. Neuron development is upregulated, and neuron death is downregulated with APC treatment (Figure 4A).

We found that astrocytes, endothelial cells, and neurons play roles in inflammatory response in 5xFAD mice. Furthermore, many interleukin processes are upregulated across all cell-types in 5xFAD mice (Figure 4B). These inflammatory processes are well implicated with AD progression and receive broad downregulation in all cell-types of APC treated mice. Interleukin-1 beta is upregulated in astrocyte, endothelial cells, and neurons of AD mice. Astrocytes, endothelial cells, oligodendrocytes, and OPCs of APC treated mice contribute to the downregulation of interleukin-1 beta production (Figure 4B). The production of interleukin-2 and interleukin-10 in 5xFAD mice is implicated with endothelial cells, neurons, and oligodendrocytes. The downregulation of interleukin-2 and interleukin-10 following APC treatment are handled by the same production cell-types in 5xFAD (Figure 4B). Every cell-type was found to participate in the production of interleukin-6 in 5xFAD mice and following APC treatment, every cell-type contributed towards the downregulation of interleukin-6 (Figure 4B).

Microglial cell activation in 5xFAD mice were found to be upregulated in the microglia and OPCs. It appears that every cell-type except oligodendrocytes contributes to downregulating the microglial activation found in APC treated 5xFAD mice (Figure 4C). Myeloid cell activation in 5xFAD mice were upregulated in neurons and oligodendrocytes and downregulated in astrocytes, endothelial cells, and oligodendrocytes in APC treatment (Figure 4C). Amyloid fibril formation and amyloid-beta clearance is upregulated in 5xFAD mice in microglia, neurons, and OPCs and microglia, oligodendrocytes, and OPCs respectively (Figure 4C). Focal adhesion in 5xFAD mice is downregulated in microglia; all cell-types except for astrocytes upregulate focal adhesion assembly in APC treatment (Figure 4C).

### Administration of APC attenuates the Aβ burden and improves cognitive function in AD mice

**Figure 5:**
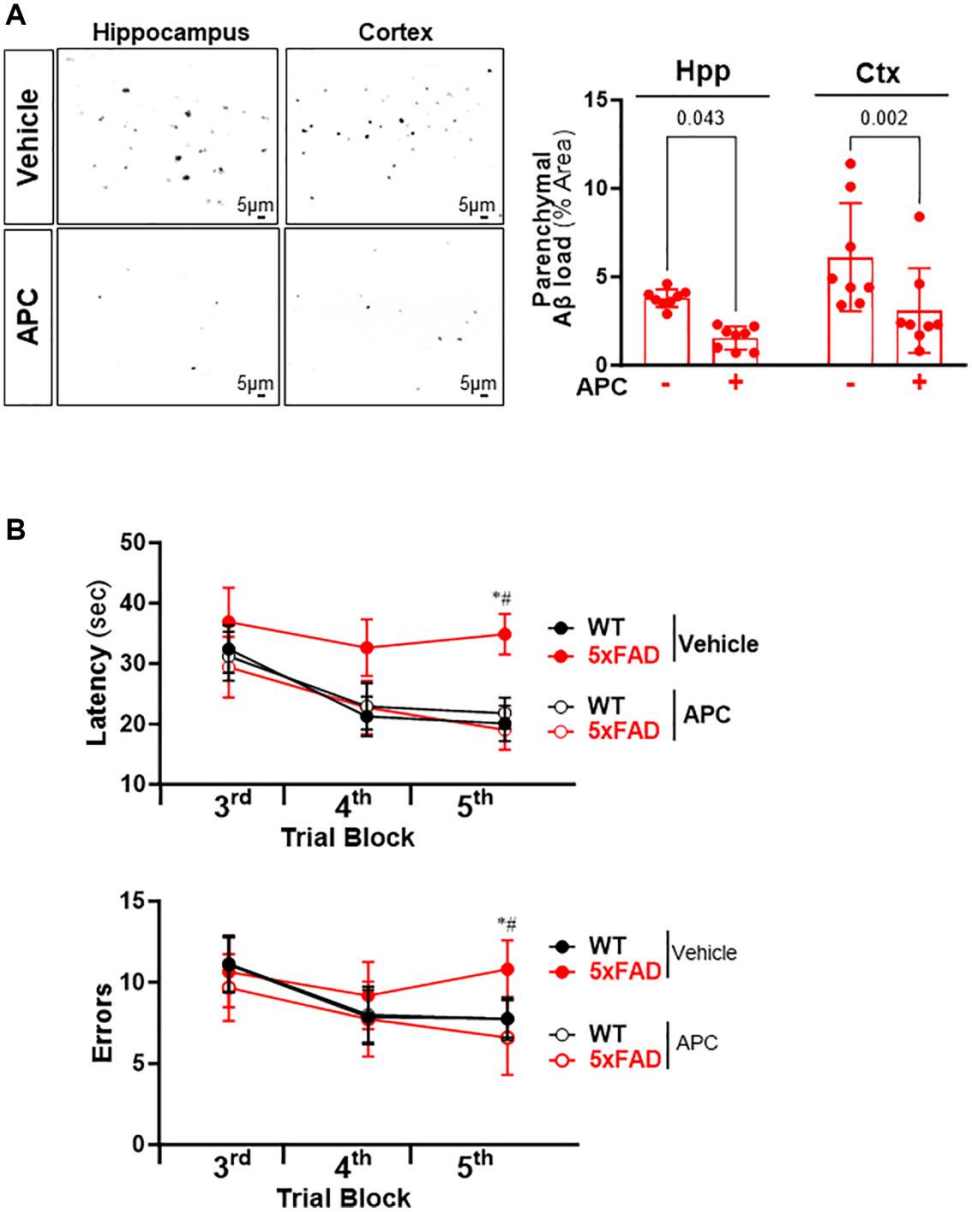
(**A**) Representative images and quantification of Aβ stained with thioflavin S in the hippocampus and cortex of 5xFAD mice treated with or without APC. (**B**) Radial arm water maze test showed the latencies to hidden platform and the errors happens in arms in WT and 5xFAD mice. *N* = 8, ^*^p < 0.05, 5xFAD-Vehicle vs. WT-Vehicle; ^#^*p* < 0.05, 5xFAD-APC vs. 5xFAD-Vehicle.

To determine whether APC treatment can reverse the amyloid plaques aggravation in AD, the 5xFAD and C57BL/6 WT mice (10 weeks old) were treated with recombinant APC daily (100 μg/kg/d i.p.) or vehicle (saline) for 5 months. We examined whether administration of recombinant APC influences the development of Aβ pathology in AD. The results demonstrated that APC treatment effectively inhibited the Aβ burden in both hippocampus and cortex (Figure 5A). Compared with vehicle, APC treatment reduced load by 53% in hippocampus and 50% in cortex, it suggests that APC efficiently delay the development of Aβ pathology and amyloid angiopathy. Considering the Aβ aggregation devastating cognitive function, we further analyzed the effects of APC treatment on the hippocampal-dependent spatial memory function with radial arm water maze. The results showed that APC treatment normalized the performance of 5xFAD mice on spatial learning and memory ability in the radial-arm water maze test (Figure 5B). Consistent with Aβ pathology, APC treatment benefits the cognitive function in AD model mice. These data indicated that APC exerts potent neuroprotective activity against AD pathology.

## DISCUSSION

The incidence of AD continues to grow and remains as the most common neurodegenerative condition [5]. Activated Protein C (APC) treatment has recently been proven to positively treat ALS and stroke in human trials [13, 14]. The neuroprotective effects of APC have been studied as potential future therapeutics for AD [12, 24]. Using single-cell RNA sequencing and bioinformatic analysis, we analyze the effects of APC treatment on AD transgenic mice. To best test the impact on AD pathophysiology, we experimented with APC on 5xFAD mice, furthering the research performed in the field [12]. 5xFAD mice exhibit many behavioral and physiological symptoms parallel to human AD including amyloid deposits, increased aggression, and depressive behavior, decreased social interactions and sleep [25].

Initial bioinformatic analysis of cell populations in our samples reveal drastic changes in the physiology of AD mice. Over half of 5xFAD sample contained microglia compared to just over 30% of the WT sample. Previous research indicates that the Aβ deposits trigger neuroinflammation and microglial activation leading to eventual metabolic reprogramming [26]. APC treatment sample uncovers a large decrease in the microglial population suggesting reversal of the immune response towards Aβ. Recent research utilizing APC has found it’s antiinflammation properties and reduction of microglial activation in ocular inflammation, these mechanisms most likely are implicated in the results we observe in our study [27]. We note that *Cx3cr1*, a marker gene used for microglial cell annotation was expressed in all samples. Deficiency of *Cx3cr1* has shown increased Aβ pathology as well as cognitive decline [28], 5xFAD mice did not show a decrease in *Cx3cr1* expression. During the cell-type annotation process we also encountered a substantial overlap of astrocyte and neurons, this has been observed in other studies have found the sharing of transcriptomic signatures between these cell-types [29]. This close transcriptional pattern made differentiating astrocyte and neuron populations challenging and downstream testing shows yielded related results. Ultimately, we find that APC treatment notably increases the population of every cell-type compared to no treatment and larger proportions of endothelial cells, neurons, and oligodendrocytes were recorded compared to WT. These extensive alterations of cell populations by APC treatment demonstrate significant impact towards AD progression.

The differential expression testing revealed that *C1qa*, *C1qb*, and *C1qc* are widely upregulated in 5xFAD. We observed downregulation of these genes by APC in every cell type except for microglia. Studies have proven that microglia are the dominant source of *C1q* in the murine brain [30]. Though inhibition of *C1q* has known to reduce the number of microglia [31, 32], *C1q* likely is not solely involved in microglial reduction due the expression level of *C1q* not exceedingly changing APC treated microglia. *C1qc* specifically when downregulated has been found to impact other microglial activation genes *Lpl*, *Lyz2*, and *Ccl4* [32]; all of which we also observe as being downregulated after APC treatment especially in microglia. The astrocyte population notably expresses two DEGs that are modulated by APC treatment. *C4b* expression was found to be greater in 5xFAD mice and is considered a biomarker for AD in humans [33]. The expression of *C4b* in human AD has confirmed the inflammation hypothesis regarding AD [34]. We observed a downregulation of *C4b* in APC treatment, while further study is needed, the anti-inflammatory properties of APC seem to mediate *C4b* levels in the brain. Additionally, the exclusive differential expression of *Gfap* in astrocytes is compelling. Like *C4b*, we observe a downregulation of *Gfap* with APC treatment. *Gfap* is implicated in astrocytes of neurodegenerative diseases [35]. Furthermore, *Gfap* also serves as a biomarker for AD in humans [36–40]. Knockout of *Gfap* in AD neuropathies has been shown to improve physiological decline and as a result has been a novel therapeutic target [41]. Continued research into the physiological impact of *Gfap* is needed as it could point towards glial activation in AD progression which we observe in 5xFAD mice [42].

APC treatment shows return to near WT levels of expression of *Cd63* and *Cd68*. Both genes are implicated and found to be expressed in AD models, however the mechanisms these genes impact is unclear in AD progression [43–45]. It is unknown if the downregulation of these genes is beneficial in the treatment of AD. The downregulation of the cathepsin family of genes by APC shows tremendous modulation of inflammatory symptoms from AD. Cathepsin B (*Ctsb*) is exceptionally recognized in not only in AD but in other brain disorders such as traumatic brain injury (TBI) [46]. Particularly, microglial *Ctsb* has been recognized to actively progress inflammatory disease and aging in the brain [47]. Moreover, *Ctsb* has been found to be upregulated in the serum of AD patients and is considered a biomarker of AD [48]. Other members of the cathepsin family such as *Ctsd* and *Ctss* have been found to play roles in amyloidosis and neuroinflammation in relation to AD as well as degrade the BBB [49, 50]. In 5xFAD transgenic mice, *Ctsb* has been found to be generally upregulated globally [46, 51]. It is believed that *Ctsb* plays a major role in lysosomal leakage and is a powerful lysosomal protease leading to neurodegeneration observed in brain disorders and aging [52]. Regarding Aβ production, *Ctsb* is thought to interact with the β-secretase site of amyloid precursor protein (APP) [53]. Previous study of *Ctsb* in AD and other neurological disorders have proven that *Ctsb* knockout (KO) transgenic mice as well as inhibition of *Ctsb* have improved behavioral deficits [54, 55]. APC treatment has previously been shown to directly impact the β-secretase pathway and the broad downregulation of *Ctsb* further implicates APC as a plausible method of mediating AD pathology and symptoms [12].

Coinciding with the cathepsin family of genes in microglia, *Cst7* is also implicated in the neuroinflammation that occurs with Alzheimer’s and prion disease [56, 57]. Expression of *Cst7* is notably expressed in microglia of AD pathology and has recently been considered a sex-dependent indicator for AD [58, 59]. *Cst7* expressing microglia tend to surround Aβ in the brain and increase inflammation [56]. The significant decrease in microglial *Cst7* by APC treatment may prove a strong decrease in Aβ presence in the brain and consequently a decrease in the population of microglia and neuroinflammation. Though this decreased expression may only prove to be beneficial to males as knockout of Cst7 in males decreases pro-inflammatory mediators while females increased pro-inflammatory and endolysosomal expression in microglia [58]. Further important AD risk genes were also found to be upregulated in 5xFAD mice and later downregulated by APC treatment. *Irf8* and *Hexb* notably have been researched as therapeutics for AD with inhibition/deletion of the genes being beneficial [60, 61]. It was proven that *Irf8* is involved in microglial activation in response to Aβ and leads to interleukin-1β inflammation [61]. The downregulation of *Irf8* by APC treatment might provide further rationale towards the decreased microglial population and mediation of AD symptoms. *Hexb* was also downregulated in multiple cell-types after APC treatment. Compared to knock-in *Hexb* mice, *Hexb* heterozygotic mice showed improved behavior and decrease in Aβ deposition [60]. Though *Hexb* knockout can be detrimental [62], the modulation of its expression may prove to be useful in AD treatment. Several microglial genes were also found to be regulated by APC treatment. Many DEGs that were found exclusively in microglia have been noted in neurodegeneration and some in specific response to Aβ known as disease-associated microglia (DAM). *Axl*, *Ccl3*, *Ccl4*, *Ccl6*, *Csf1*, *Igf1*, *Itgax*, *Lpl*, *Lilrb4*, *Spp1* were all found exclusively in microglia and known to upregulated in AD [21, 45, 63, 64]. Though *Apoe*, *Cd63*, *Fcer1g*, *Grn*, *Laptm5*, *Lgals3*, and *Timp2* were found differentially expressed in other cell-types, their microglial expression has been found linked to Aβ as well [45, 63–66]. APC treatment shows incredible downregulation of almost all these genes indicating the decreased Aβ deposition found in APC treated 5xFAD mice [12].

Our study comprehensively quantified the transcriptomes in different cell types of mouse brains, including wild-type mice, 5xFAD mice, and 5xFAD mice treated with APC. To further identify differences in the genome of wild type, 5xFAD, and APC-treated 5xFAD mice, the study of a genomic variant at a single base position is necessary. We believe that in the future, completing a deep single-cell RNA-seq approach will be essential. The use of different sequencing technologies that allow for the collection of whole genomes would be immensely helpful in advancing our study. Additionally, other omics data such as metabolomics and proteomics study of APC treatment on AD would be able to provide further observation of the physiological changes occurring. We plan to do further biochemical experimentation as well as investigate multiple DEGs uncovered in knockout mice. In future single-cell RNA seq studies, we would also like to collect brain tissue along the 5-month APC treatment course and observe transcriptomic changes that occur during treatment. In the future, we plan to obtain this derivative and experiment with not only 5xFAD transgenic mice but other strains such as those expressing tau physiology. APC treatment in AD Tg2576 transgenic mice has also been found to inhibit Aβ production by a different mechanism and improve memory deficits through promotion of α-secretase [24]. This discrepancy could be attributed to the use of Tg2576 versus 5xFAD transgenic mice. Sc-RNA seq of APC-treatment on Tg2576 mice may be useful for understanding further AD mechanisms that are impacted by APC treatment.

## MATERIALS AND METHODS

### Selection of individual and sample preparation

Both male and female C57BL/6 wild type mice and 5xFAD C57BL/6 mice were supplied from Jackson Laboratory (Bar Harbor, ME, USA). Three biological replicates (whole brain tissue) were used per sample. All animal protocols used for this study were approved by the Institutional Animal Care and Use committee of the University of South Florida and comply to the NIH guide for the care and use of laboratory animals.

At 2 months of age, recombinant WT murine APC (100 μg/kg) was injected daily via i.p. for 5 months, collecting brain tissue in 5xFAD and WT mice. Four brain tissue samples were subject to dissociation to be sent for sequencing: WT, WT with APC, 5xFAD, 5xFAD with APC. Mice’s brains were excised and rinsed with PBS, subsequently minced with Miltenyi Biotec Adult Brian Dissociation kit for mouse and rat following the manual protocol. First, mice brain samples were minced into 2 mm by 2 mm pieces with Enzyme Mix 1, after adding Enzyme Mix 2, samples were incubated using program 37C_ABDK_01 with gentleMACS Octo Dissociator. After the completion of the program, FBS was added to stop the reaction, and the suspension was filtered by a 70 μm MACS SmartStrainer on top of a Falcon 40 μm cell strainer. Cells were pelleted at 600 × g for 5 mins at 4ºC for debris removal. Second, cold PBS and Debris Removal Solution were used following the manual protocol to remove the debris. Third, Red blood cell lysis was carried out with PEB buffer and Red Blood Cell Lysis Solution to remove erythrocytes. At last, after samples were washed with cold PBS with 0.05% BSA buffer three times and filtered through Flowmi Top Strainer, single-cell samples were counted with an automated CellCounter.

### Analysis of scRNA seq data

Cells were processed using the Chromium Next Gem Single Cell 3′ Reagent V3.1 kit from 10x Genomics. A total of 10,000 cells per sample were loaded into a Chip G for GEM generation. Reverse transcription, barcoding, complementary DNA amplification and purification for library preparation were performed according to protocol. Sequencing was performed on a NovaSeq 6000 platform (Illumina) targeting 100,000 reads per cell and received as Sanger/Illumina 1.9 encoded FASTQ files. Quality scores (>28) of each FASTQ file were confirmed by *FASTQC 0.11.15* (Babraham Bioinformatics, Cambridge). Processing of raw FASTQ files for bioinformatic analysis was completed by the *10x Genomics Cellranger* [67] platform using the *10x Genomics* mm10 reference genome using default parameters for the *count* function for each sample. Raw and processed data can be accessed under the GEO accession GSE227157.

The *Seurat* 4.0 [68] package in Rstudio 2022.12.0 + 353 IDE (R Core Team, 2022 and Posit Team, 2022) was applied to normalize and dimensionally reduce data. Raw samples contained ~21,000 features. Samples were log-normalized and top variable features were determined by the “vst” selection method for the top 2000 variable features with 30 dimensions from the canonical correlation analysis (CCA). The Seurat integration protocol was selected for this study to compare datasets while retaining biological variability and minimizing technical differences. The various tools provided by Seurat are recommended to correct for batch effects in scRNA-seq data [69]. The integration protocol was used for “anchoring” cells between datasets (batches) utilizing mutual nearest neighbors (MMN) approach [19]. These “anchors” group cells based on similar expression profiles and therefore likely to be of the same cell-type to be identified after dimensional reduction and clustering. Integration code is available in Supplementary File 1 (Integration.txt).

### Cell clustering

The integrated dataset contains ~24,000 features and the top 2000 variable features which were scaled using the default linear model and a PCA reduction. Based on the amount of standard deviation (Supplementary Figure 3A), reduction to 10 principal components were used for the remaining downstream analysis to capture most variation within the dataset. UMAP was run on the top 10 principal components (PCs) (Supplementary Figure 3B). Clustering resolution (0.3) was determined by visually examining the incoming proportion of cells as resolution increased (Supplementary Figure 3B) using *Clustree* 0.5.0 [70].

### Cell type annotation

Utilizing the *(FindConservedMarkers)* function in *Seurat*, we identified conserved marker genes in each unsupervised cluster to use for cell-type annotation. This function outputs a data frame containing all genes expressed in each sample and their corresponding Log_2_FC and adjusted *p*-values. Adjusted *p*-values are generated in *Seurat* by statistically comparing expression with other genes in the assay and performing Bonferroni correction [71]. These conserved genes were found to be expressed in each sample and the top three genes were used for annotation (Figure 1C).

Cross-analysis with the *Human Protein Atlas* [72] was used to verify marker genes for annotation. Clusters that did not highly express (Log_2_FC >3) canonical marker genes for Astrocytes, Endothelial cells, Microglia, Neurons, Oligodendrocytes, or Oligodendrocyte Precursor Cells (OPC) were not included for further downstream analysis to retain accuracy of annotations. Cell-type annotation code available in Supplementary File 2 (Cell-Annotation.txt).

### Differentially expressed genes analysis

The *Seurat* vignette for differential expression testing was followed for differentially expressed gene (DEG) identification. In our testing parameters, two samples within the dataset were isolated compared with each cell-type at a time. The RNA data assay was used in contrast to the integrated assay as the RNA assay retains the original expression profiles of cells prior to “correction” that occurs during the integration protocol [19]. The original sample identities of the cells were set as the identities in the *(FindMarkers)* function with cell-type set as the subset identity. This function outputs a data frame containing all differentially expressed genes between the two identities set and their corresponding Log_2_FC and adjusted *p*-values. Adjusted *p*-values are generated in *Seurat* by statistically comparing expression with other genes in the assay and performing Bonferroni correction [71]. All genes possessing an adjusted *p*-value of greater than or equal to 0.05 were omitted from further downstream analysis. Lists of significant DEGs (Log2FC > ± 1, adj. *p*-value < 0.05) per cell-type available in Excel format in Supplementary Files 3 and 4 (DEG-Dataset 1_5xFAD vs. WT.xlsx, DEG-Dataset 2_5xFAD APC vs. 5xFAD Vehicle.xlsx).

Bulk RNA-sequencing data was retrieved from the GEO accession GSE140286 [73]. Utilizing data from 5xFAD and WT mice at 6 months of age that aligns closely to the 7-month age of our mice. Differential expression testing between 6-month-old 5xFAD and WT mice bulk RNA-seq data was completed using *Limma 3.16* [74] in RStudio 2022.12.0 + 353 IDE (R Core Team, 2022 and Posit Team, 2022). Enrichment data was processed by *EnrichR*. A data frame containing all associated GO biological processes and corresponding adjusted *p*-values is produced from the data obtained from the differential expression testing performed previously. *EnrichR* produces Benjamini-Hochberg adjusted *p*-values [75, 76]. All GO terms possessing an adjusted *p*-value of greater than or equal to 0.05 were excluded and terms representing developmental alterations and immune responses in the brain were selected. Differential expression testing and enrichment code available in Supplementary File 5 (DE_Enrichment.txt).

### Amyloid plaques deposit staining

Heparin IV for anticoagulation was given by intraperitoneal injection with 1,000 units/kg 10 min before the experiment [77]. 5xFAD and C57BL/6 WT mice (10 weeks old) were treated with recombinant APC daily (100 μg/kg/d i.p.) or vehicle (saline) for 5 months. The experimental mice underwent anesthesia with 2–3% isoflurane and 100% O_2_. The mice were transcardially perfused with ice-cold PBS. Brains were rapidly removed and fixed with 4% paraformaldehyde overnight at 4°C. Subsequently, the hemisphere was subjected to dehydration with 10, 20, and 30% sucrose and then embedded in a cutting temperature compound (Tissue-Tek). The fixed brains were sectioned at 25 μm thickness setting on a cryostat and postfixes. After washing with PBS, the sections were blocked with 5% normal donkey serum (Vector Lab)/0.1% Triton-X/PBS for 1 h and incubated with mouse anti-human 6E10 amyloid plaque antibody (Biolegend, CA, USA) diluted in blocking solution overnight at 4°C. After three PBS washes, sections were incubated with secondary antibodies in diluted blocking solution for 1 h at room temperature. Each stained section was incubated in 500 μM of Tiofavin S (TS, Sigma-Aldrich, MO, USA) dissolved in 50% ethanol for 7 min for TS double-staining. Finally, sections were washed with PBS three times and mounted onto slides with mounting medium, and observed on an SP8 confocal microscope (Leica). ImageJ was used to quantify the amyloid plaques load in the hippocampus and cortex.

### Radial arm water maze behavior test

The experimental group mice were put into the behavior room in darkness 1 h prior to the start of the experiments. For 15 consecutive days, the experiment was started around the same time, and each mouse was in the same order. This experiment was carried out in the dark. WT or 5xFAD mice were put into the water maze one at a time at the starting arm. Each day, the platform was placed at the end of the goal arm. For each trial, mice were placed at the center of the starting arm, facing forward. As it started swimming, the experimenter timed for 60 seconds and stopped timing once it reached and climbed up the platform. Errors were counted as: (1) each time the mouse entered the wrong arm, the experimenter gently grabbed its tail and pulled it back to its starting position; (2) if the mouse stayed in the center and did not enter any arm for 15 seconds; (3) if the mouse entered the goal arm without climbing up the platform for 15 seconds; (4) if the mouse did not reach the platform within 60 seconds, while only entering 1 or 2 arms continuously. After the mouse reached the platform, it was allowed to stay on the platform for 30 seconds to gain familiarity with the surrounding. For each trial, the time the mouse took to reach the platform and the number of errors it made were recorded. After the 4th trial, the mouse waited for 30 min to run the 5th trial. After each trial, the water was stirred to avoid the remaining scent that would affect the next mouse. Male and female mice were tested separately and were given 1 h in between to let the scent dissipate.

## Supplementary Materials

Supplementary Figures

Supplementary File 1

Supplementary File 2

Supplementary File 3

Supplementary File 4

Supplementary File 5
