# Supplementary material for: Single-Cell RNA-seq reveals transcriptomic modulation of Alzheimer’s disease by activated protein C
Source: Aging (Albany NY). 2024 Feb 21;16(4):3137–59. doi: 10.18632/aging.205624 (PMC10929801; doi:10.18632/aging.205624)
Supplement: Supplementary Figures [file aging-16-205624-s001.pdf]

SUPPLEMENTARY FIGURES

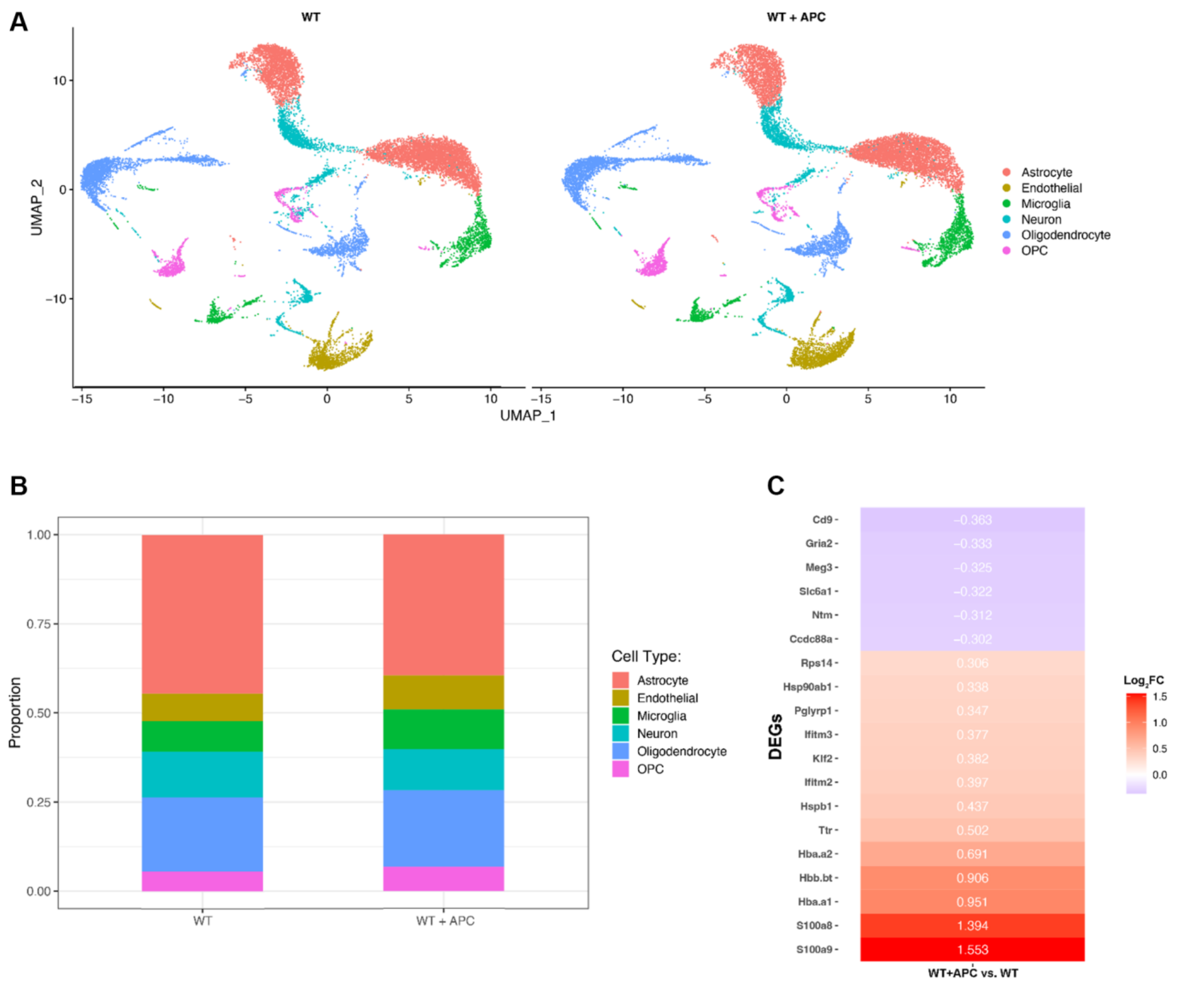

Supplementary Figure 1. (**A**) UMAP dimensional plot of control dataset: WT + APC versus WT. (**B**) Proportion of each cell-type observed in each sample of the control dataset. (**C**) Heatmap of all DEGs globally in the control dataset (p-value < 0.05).

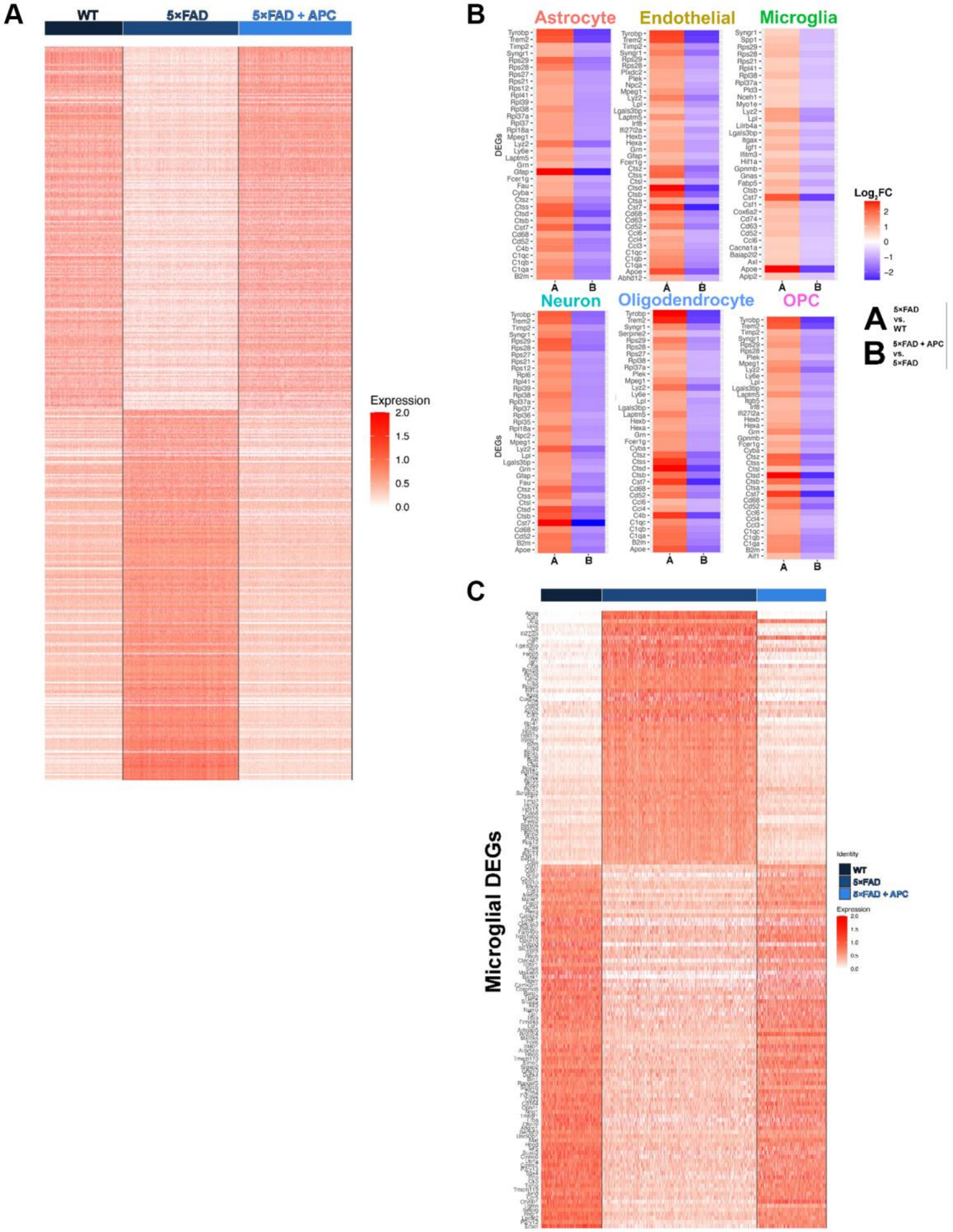

Supplementary Figure 2. (**A**) Global heatmap of all DEGs in the primary dataset (~500 DEGs) (p-value < 0.05). (**B**) Heatmap of top ~30 DEGs found in every cell-type in the primary integrated dataset. (**C**) Heatmap of all DEGs found within the microglial subset (p-value < 0.05).

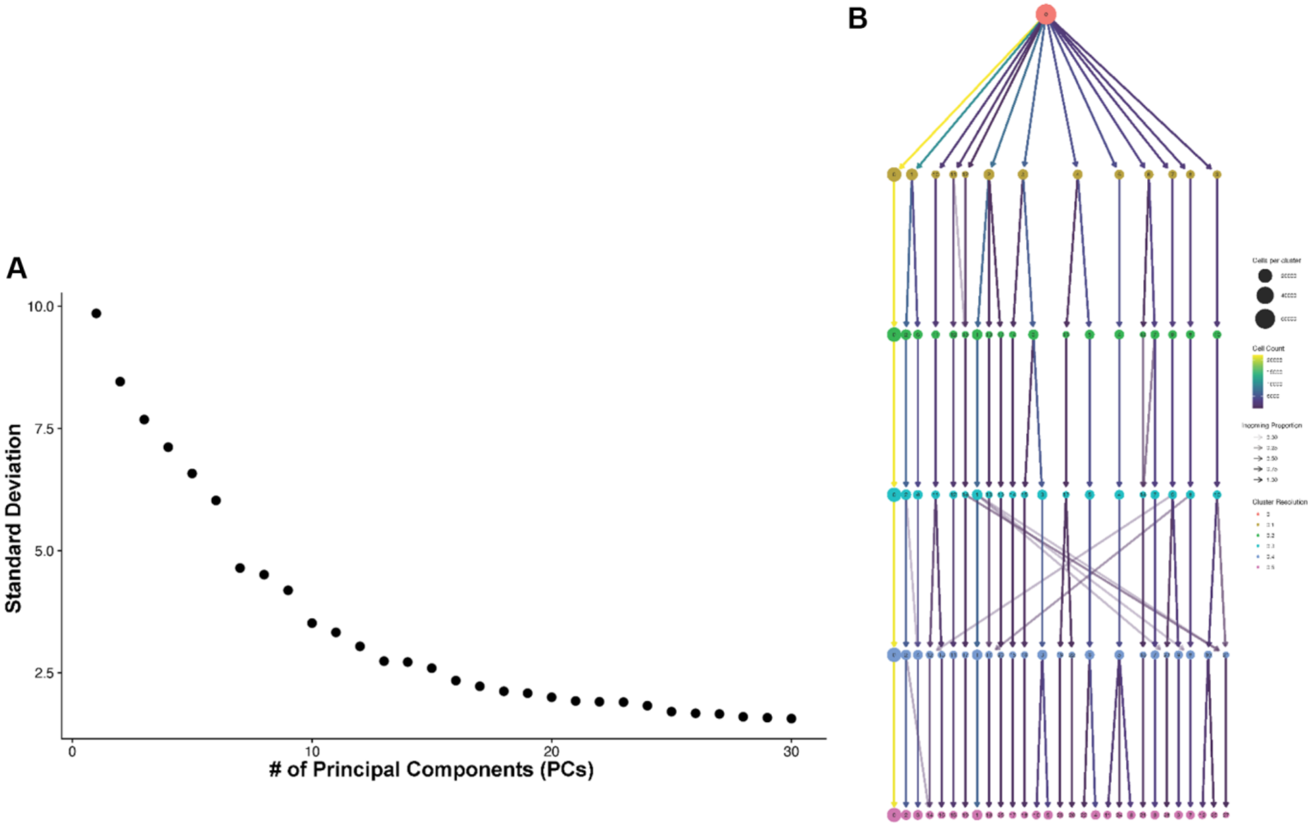

Supplementary Figure 3. (**A**) Elbow plot of variance per PC in the primary integrated dataset. (**B**) Cluster Tree of unsupervised clustering performed by Seurat.
