# Supplementary material for: Single-Cell RNA-seq reveals transcriptomic modulation of Alzheimer’s disease by activated protein C
Source: Aging (Albany NY). 2024 Feb 21;16(4):3137–59. doi: 10.18632/aging.205624 (PMC10929801; doi:10.18632/aging.205624)
Supplement: Supplementary File 1 [file aging-16-205624-s002.pdf]

Supplementary File 1. (Integration.txt): R code used to complete the *Seurat* integration dataset that combines the wildtype, 5xFAD, and 5xFAD + APC samples.

```
library(Seurat)
library(patchwork)
library(dplyr)
library(clustree)
# Process integrated dataset, 10 PCs
treatment.integrated_scale <- ScaleData(treatment.integrated)
treatment.integrated_scale <- RunPCA(treatment.integrated_scale,
                                     npcs = 10,
                                     approx = FALSE)
treatment.integrated_scale <- RunUMAP(treatment.integrated_scale,reduction = "pca", features = NULL,
                                      dims = 1:10)
treatment.integrated_scale <- FindNeighbors(treatment.integrated_scale,
                                            dims = 1:10)
#Determine Clustering resolution
treatment.integrated_scale_cluster <- FindClusters(treatment.integrated_scale,
                                           resolution = c(0,0.1,0.2,0.3,0.4,0.5))
clustree(treatment.integrated_scale_cluster, prefix = "integrated_snn_res.")
#Choose Clustering resolution
treatment.integrated_scale <-FindClusters(treatment.integrated_scale, resolution = 0.3)
rm(treatment.integrated_scale_cluster)
#Find conserved markers for every cluster
conserved.marker.0 <- FindConservedMarkers(treatment.integrated_scale, only.pos=TRUE, grouping.var =
"treatment", logfc.threshold=1,
                                         ident.1="0")
conserved.marker.1 <- FindConservedMarkers(treatment.integrated_scale, only.pos=TRUE, grouping.var =
"treatment", logfc.threshold=1,
                                           ident.1="1")
conserved.marker.2 <- FindConservedMarkers(treatment.integrated_scale, only.pos=TRUE, grouping.var =
"treatment", logfc.threshold=1,
                                           ident.1="2")
conserved.marker.3 <- FindConservedMarkers(treatment.integrated_scale, only.pos=TRUE, grouping.var =
"treatment", logfc.threshold=1,
                                           ident.1="3")
conserved.marker.4 <- FindConservedMarkers(treatment.integrated_scale, only.pos=TRUE, grouping.var =
"treatment", logfc.threshold=1,
                                           ident.1="4")
conserved.marker.5 <- FindConservedMarkers(treatment.integrated_scale, only.pos=TRUE, grouping.var =
"treatment", logfc.threshold=1,
                                           ident.1="5")
```

```
conserved.marker.6 <- FindConservedMarkers(treatment.integrated_scale, only.pos=TRUE, grouping.var =
"treatment", logfc.threshold=1,
                                          ident.1="6")
conserved.marker.7 <- FindConservedMarkers(treatment.integrated_scale, only.pos=TRUE, grouping.var =
"treatment", logfc.threshold=1,
                                          ident.1="7")
conserved.marker.8 <- FindConservedMarkers(treatment.integrated_scale, only.pos=TRUE, grouping.var =
"treatment", logfc.threshold=1,
                                          ident.1="8")
conserved.marker.9 <- FindConservedMarkers(treatment.integrated_scale, only.pos=TRUE, grouping.var =
"treatment", logfc.threshold=1,
                                          ident.1="9")
conserved.marker.10 <- FindConservedMarkers(treatment.integrated_scale, only.pos=TRUE, grouping.var =
"treatment", logfc.threshold=1,
                                          ident.1="10")
conserved.marker.11 <- FindConservedMarkers(treatment.integrated_scale, only.pos=TRUE, grouping.var =
"treatment", logfc.threshold=1,
                                          ident.1="11")
conserved.marker.12 <- FindConservedMarkers(treatment.integrated_scale, only.pos=TRUE, grouping.var =
"treatment", logfc.threshold=1,
                                           ident.1="12")
conserved.marker.13 <- FindConservedMarkers(treatment.integrated_scale, only.pos=TRUE, grouping.var =
"treatment", logfc.threshold=1,
                                           ident.1="13")
conserved.marker.14 <- FindConservedMarkers(treatment.integrated_scale, only.pos=TRUE, grouping.var =
"treatment", logfc.threshold=1,
                                           ident.1="14")
conserved.marker.15 <- FindConservedMarkers(treatment.integrated_scale, only.pos=TRUE, grouping.var =
"treatment", logfc.threshold=1,
                                           ident.1="15")
conserved.marker.16 <- FindConservedMarkers(treatment.integrated_scale, only.pos=TRUE, grouping.var =
"treatment", logfc.threshold=1,
                                           ident.1="16")
conserved.marker.17 <- FindConservedMarkers(treatment.integrated_scale, only.pos=TRUE, grouping.var =
"treatment", logfc.threshold=1,
                                           ident.1="17")
conserved.marker.18 <- FindConservedMarkers(treatment.integrated_scale, only.pos=TRUE, grouping.var =
"treatment", logfc.threshold=1,
                                           ident.1="18")
```

```
conserved.marker.19 <- FindConservedMarkers(treatment.integrated_scale, only.pos=TRUE, grouping.var =
"treatment", logfc.threshold=1,
                                            ident.1="19")
#Form new Seurat object with clusters expressing strong conserved markers
ywt_fad_apc <- subset(treatment.integrated_scale, idents = c(0,1,2,3,5,6,7,8,10,11,13,14,15,16,17,18,19))
#Annotate Cell-types in order of subset idents
new.cluster.ids <- c("Microglia","Oligodendrocyte","Astrocyte","Endothelial","Oligodendrocyte","Neuron",

"OPC","Oligodendrocyte","Oligodendrocyte","OPC","Oligodendrocyte","Neuron","Endothelial",
                                        "Oligodendrocyte","Oligodendrocyte","OPC","Oligodendrocyte")
names(new.cluster.ids) <- levels(ywt_fad_apc)
ywt_fad_apc <- RenameIdents(ywt_fad_apc, new.cluster.ids)
#Place Cell-types in alphabetical order
levels(ywt_fad_apc) <- c("Astrocyte","Endothelial", "Microglia", "Neuron", "Oligodendrocyte", "OPC")
```
