# Supplementary material for: Single-Cell RNA-seq reveals transcriptomic modulation of Alzheimer’s disease by activated protein C
Source: Aging (Albany NY). 2024 Feb 21;16(4):3137–59. doi: 10.18632/aging.205624 (PMC10929801; doi:10.18632/aging.205624)
Supplement: Supplementary File 2 [file aging-16-205624-s003.pdf]

Supplementary File 2. (Cell-Annotation.txt): R code used to identify the types of cells contained in the integrated *Seurat* dataset across wildtype, 5xFAD, and 5xFAD + APC samples.

```
library(enrichR)
#Find all DEGs between 5xFAD+APC and 5xFAD regardless of cell-type
global1 <- FindMarkers(ywt_fad_apc, ident.1 = "apc", ident.2 = "fad", group.by = "treatment", assay =
"RNA")
#Find DEGs in each cell-type between 5xFAD+APC and 5xFAD
astrocyte1 <-FindMarkers(ywt_fad_apc, ident.1 = "apc", ident.2 = "fad", group.by = "treatment",
                  assay = "RNA", subset.ident = "Astrocyte")
endothelial1 <-FindMarkers(ywt_fad_apc, ident.1 = "apc", ident.2 = "fad", group.by = "treatment",
                   assay = "RNA", subset.ident = "Endothelial")
microglia1 <-FindMarkers(ywt_fad_apc, ident.1 = "apc", ident.2 = "fad", group.by = "treatment",
                   assay = "RNA", subset.ident = "Microglia")
neuron1 <-FindMarkers(ywt_fad_apc, ident.1 = "apc", ident.2 = "fad", group.by = "treatment",
                   assay = "RNA", subset.ident = "Neuron")
oligodendrocyte1 <-FindMarkers(ywt_fad_apc, ident.1 = "apc", ident.2 = "fad", group.by = "treatment",
                   assay = "RNA", subset.ident = "Oligodendrocyte")
opc1 <-FindMarkers(ywt_fad_apc, ident.1 = "apc", ident.2 = "fad", group.by = "treatment",
                    assay = "RNA", subset.ident = "OPC")
#Find all DEGs between 5xFAD and WT regardless of cell-type
global2 <- FindMarkers(ywt_fad_apc, ident.1 = "fad", ident.2 = "ywt", group.by = "treatment", assay =
"RNA")
#Find DEGs in each cell-type between 5xFAD and WT
astrocyte2 <-FindMarkers(ywt_fad_apc, ident.1 = "fad", ident.2 = "ywt", group.by = "treatment",
                  assay = "RNA", subset.ident = "Astrocyte")
endothelial2 <-FindMarkers(ywt_fad_apc, ident.1 = "fad", ident.2 = "ywt", group.by = "treatment",
                   assay = "RNA", subset.ident = "Endothelial")
microglia2 <-FindMarkers(ywt_fad_apc, ident.1 = "fad", ident.2 = "ywt", group.by = "treatment",
                   assay = "RNA", subset.ident = "Microglia")
neuron2 <-FindMarkers(ywt_fad_apc, ident.1 = "fad", ident.2 = "ywt", group.by = "treatment",
                   assay = "RNA", subset.ident = "Neuron")
oligodendrocyte2 <-FindMarkers(ywt_fad_apc, ident.1 = "fad", ident.2 = "ywt", group.by = "treatment",
                   assay = "RNA", subset.ident = "Oligodendrocyte")
opc2 <-FindMarkers(ywt_fad_apc, ident.1 = "fad", ident.2 = "ywt", group.by = "treatment",
                    assay = "RNA", subset.ident = "OPC")
#Enrichment of 5xFAD+APC and 5xFAD Differential expression data
setEnrichrSite("Enrichr")
listEnrichrDbs()
dbs <- listEnrichrDbs()
dbs <- c("GO_Biological_Process_2021")
#Astrocyte Upregulated, Removing all terms with adjusted p-value >0.05
```

```
Astro <- astrocyte1
Astrou <- Astro[Astro$avg_log2FC >=0, ]
eAstrou <- enrichr(Astrou$X,dbs)
threshold0 <- eAstrou$GO_Biological_Process_2021$Adjusted.P.value < 0.05
eAstrou$GO_Biological_Process_2021$threshold <- threshold0
eAstrou$GO_Biological_Process_2021 <-
eAstrou$GO_Biological_Process_2021[!grepl("FALSE",eAstrou$GO_Biological_Process_2021$threshold),]
remove.col <- "threshold"
eAstrou$GO_Biological_Process_2021 <-
eAstrou$GO_Biological_Process_2021[,!names(eAstrou$GO_Biological_Process_2021) %in% remove.col, drop = F]
#Enriched terms in eAstrou$GO_Biological_Process_2021
#Astrocyte Downregulated, Removing all terms with adjusted p-value >0.05
Astrod <- Astro[Astro$avg_log2FC <=0, ]
eAstrod <- enrichr(Astrod$X,dbs)
threshold0 <- eAstrod$GO_Biological_Process_2021$Adjusted.P.value < 0.05
eAstrod$GO_Biological_Process_2021$threshold <- threshold0
eAstrod$GO_Biological_Process_2021 <-
eAstrod$GO_Biological_Process_2021[!grepl("FALSE",eAstrod$GO_Biological_Process_2021$threshold),]
remove.col <- "threshold"
eAstrod$GO_Biological_Process_2021 <-
eAstrod$GO_Biological_Process_2021[,!names(eAstrod$GO_Biological_Process_2021) %in% remove.col, drop = F]
#Enriched terms in eAstrod$GO_Biological_Process_2021
#Endothelial Upregulated, Removing all terms with adjusted p-value >0.05
Endo <- endothelial1
Endou <- Endo[Endo$avg_log2FC >=0, ]
eEndou <- enrichr(Endou$X,dbs)
threshold0 <- eEndou$GO_Biological_Process_2021$Adjusted.P.value < 0.05
eEndou$GO_Biological_Process_2021$threshold <- threshold0
eEndou$GO_Biological_Process_2021 <-
eEndou$GO_Biological_Process_2021[!grepl("FALSE",eEndou$GO_Biological_Process_2021$threshold),]
remove.col <- "threshold"
eEndou$GO_Biological_Process_2021 <-
eEndou$GO_Biological_Process_2021[,!names(eEndou$GO_Biological_Process_2021) %in% remove.col, drop = F]
#Enriched terms in eEndou$GO_Biological_Process_2021
#Endothelial Downregulated, Removing all terms with adjusted p-value >0.05
Endod <- Endo[Endo$avg_log2FC <=0, ]
eEndod <- enrichr(Endod$X,dbs)
threshold0 <- eEndod$GO_Biological_Process_2021$Adjusted.P.value < 0.05
eEndod$GO_Biological_Process_2021$threshold <- threshold0
```

```
eEndod$GO_Biological_Process_2021 <-
eEndod$GO_Biological_Process_2021[!grepl("FALSE",eEndod$GO_Biological_Process_2021$threshold),]
remove.col <- "threshold"
eEndod$GO_Biological_Process_2021 <-
eEndod$GO_Biological_Process_2021[,!names(eEndod$GO_Biological_Process_2021) %in% remove.col, drop = F]
#Enriched terms in eEndod$GO_Biological_Process_2021
#Microglia Upregulated, Removing all terms with adjusted p-value >0.05
Micro <- microglia1
Microu <- Micro[Micro$avg_log2FC >=0, ]
eMicrou <- enrichr(Microu$X,dbs)
threshold0 <- eMicrou$GO_Biological_Process_2021$Adjusted.P.value < 0.05
eMicrou$GO_Biological_Process_2021$threshold <- threshold0
eMicrou$GO_Biological_Process_2021 <-
eMicrou$GO_Biological_Process_2021[!grepl("FALSE",eMicrou$GO_Biological_Process_2021$threshold),]
remove.col <- "threshold"
eMicrou$GO_Biological_Process_2021 <-
eMicrou$GO_Biological_Process_2021[,!names(eMicrou$GO_Biological_Process_2021) %in% remove.col, drop = F]
#Enriched terms in eMicrou$GO_Biological_Process_2021
#Microglia Downregulated, Removing all terms with adjusted p-value >0.05
Microd <- Micro[Micro$avg_log2FC <=0, ]
eMicrod <- enrichr(Microd$X,dbs)
threshold0 <- eMicrod$GO_Biological_Process_2021$Adjusted.P.value < 0.05
eMicrod$GO_Biological_Process_2021$threshold <- threshold0
eMicrod$GO_Biological_Process_2021 <-
eMicrod$GO_Biological_Process_2021[!grepl("FALSE",eMicrod$GO_Biological_Process_2021$threshold),]
remove.col <- "threshold"
eMicrod$GO_Biological_Process_2021 <-
eMicrod$GO_Biological_Process_2021[,!names(eMicrod$GO_Biological_Process_2021) %in% remove.col, drop = F]
#Enriched terms in eMicrod$GO_Biological_Process_2021
#Neuron Upregulated, Removing all terms with adjusted p-value >0.05
Neuro <- neuron1
Neurou <- Neuro[Neuro$avg_log2FC >=0, ]
eNeurou <- enrichr(Neurou$X,dbs)
threshold0 <- eNeurou$GO_Biological_Process_2021$Adjusted.P.value < 0.05
eNeurou$GO_Biological_Process_2021$threshold <- threshold0
eNeurou$GO_Biological_Process_2021 <-
eNeurou$GO_Biological_Process_2021[!grepl("FALSE",eNeurou$GO_Biological_Process_2021$threshold),]
remove.col <- "threshold"
eNeurou$GO_Biological_Process_2021 <-
```

```
eNeurou$GO_Biological_Process_2021[,!names(eNeurou$GO_Biological_Process_2021) %in% remove.col, drop = F]
#Enriched terms in eNeurou$GO_Biological_Process_2021
#Neuron Downregulated, Removing all terms with adjusted p-value >0.05
Neurod <- Neuro[Neuro$avg_log2FC <=0, ]
eNeurod <- enrichr(Neurod$X,dbs)
threshold0 <- eNeurod$GO_Biological_Process_2021$Adjusted.P.value < 0.05
eNeurod$GO_Biological_Process_2021$threshold <- threshold0
eNeurod$GO_Biological_Process_2021 <-
eNeurod$GO_Biological_Process_2021[!grepl("FALSE",eNeurod$GO_Biological_Process_2021$threshold),]
remove.col <- "threshold"
eNeurod$GO_Biological_Process_2021 <-
eNeurod$GO_Biological_Process_2021[,!names(eNeurod$GO_Biological_Process_2021) %in% remove.col, drop = F]
#Enriched terms in eNeurod$GO_Biological_Process_2021
#Oligodendrocyte Upregulated, Removing all terms with adjusted p-value >0.05
Oligo <- oligodendrocyte1
Oligou <- Oligo[Oligo$avg_log2FC >=0, ]
eOligou <- enrichr(Oligou$X,dbs)
threshold0 <- eOligou$GO_Biological_Process_2021$Adjusted.P.value < 0.05
eOligou$GO_Biological_Process_2021$threshold <- threshold0
eOligou$GO_Biological_Process_2021 <-
eOligou$GO_Biological_Process_2021[!grepl("FALSE",eOligou$GO_Biological_Process_2021$threshold),]
remove.col <- "threshold"
eOligou$GO_Biological_Process_2021 <-
eOligou$GO_Biological_Process_2021[,!names(eOligou$GO_Biological_Process_2021) %in% remove.col, drop = F]
#Enriched terms in eOligou$GO_Biological_Process_2021
#Oligodendrocyte Downregulated, Removing all terms with adjusted p-value >0.05
Oligod <- Oligo[Oligo$avg_log2FC <=0, ]
eOligod <- enrichr(Oligod$X,dbs)
threshold0 <- eOligod$GO_Biological_Process_2021$Adjusted.P.value < 0.05
eOligod$GO_Biological_Process_2021$threshold <- threshold0
eOligod$GO_Biological_Process_2021 <-
eOligod$GO_Biological_Process_2021[!grepl("FALSE",eOligod$GO_Biological_Process_2021$threshold),]
remove.col <- "threshold"
eOligod$GO_Biological_Process_2021 <-
eOligod$GO_Biological_Process_2021[,!names(eOligod$GO_Biological_Process_2021) %in% remove.col, drop = F]
#Enriched terms in eOligod$GO_Biological_Process_2021
#OPC Upregulated, Removing all terms with adjusted p-value >0.05
OPC <- opc1
OPCu <- OPC[OPC$avg_log2FC >=0, ]
```

```
eOPCu <- enrichr(OPCu$X,dbs)
threshold0 <- eOPCu$GO_Biological_Process_2021$Adjusted.P.value < 0.05
eOPCu$GO_Biological_Process_2021$threshold <- threshold0
eOPCu$GO_Biological_Process_2021 <-
eOPCu$GO_Biological_Process_2021[!grepl("FALSE",eOPCu$GO_Biological_Process_2021$threshold),]
remove.col <- "threshold"
eOPCu$GO_Biological_Process_2021 <-
eOPCu$GO_Biological_Process_2021[,!names(eOPCu$GO_Biological_Process_2021) %in% remove.col, drop = F]
#Enriched terms in eOPCu$GO_Biological_Process_2021
#OPC Downregulated, Removing all terms with adjusted p-value >0.05
OPCd <- OPC[OPC$avg_log2FC <=0, ]
eOPCd <- enrichr(OPCd$X,dbs)
threshold0 <- eOPCd$GO_Biological_Process_2021$Adjusted.P.value < 0.05
eOPCd$GO_Biological_Process_2021$threshold <- threshold0
eOPCd$GO_Biological_Process_2021 <-
eOPCd$GO_Biological_Process_2021[!grepl("FALSE",eOPCd$GO_Biological_Process_2021$threshold),]
remove.col <- "threshold"
eOPCd$GO_Biological_Process_2021 <-
eOPCd$GO_Biological_Process_2021[,!names(eOPCd$GO_Biological_Process_2021) %in% remove.col, drop = F]
#Enriched terms in eOPCd$GO_Biological_Process_2021
#Clean Environment
rm(Astro, Endo, Micro, Neuro, Oligo, OPC, Astrod, Astrou, dbs,
   Endod, Endou, Microd, Microu, Neurod, Neurou, Oligod, Oligou, OPCd, OPCu, remove.col, threshold0)
```
