# Supplementary material for: Single-Cell RNA-seq reveals transcriptomic modulation of Alzheimer’s disease by activated protein C
Source: Aging (Albany NY). 2024 Feb 21;16(4):3137–59. doi: 10.18632/aging.205624 (PMC10929801; doi:10.18632/aging.205624)
Supplement: Supplementary File 5 [file aging-16-205624-s006.pdf]

Supplementary File 5. (DE_Enrichment.txt): R code used to complete *Seurat* differential expression testing within the integrated dataset as well as generate the GO biological processes enrichment using *EnrichR*.

```
library(Seurat)
library(future)
library(patchwork)
#Use future package to use multiple cores to process data, using 8 processes, 6Gb ram each
plan("multisession",workers=8)
options(future.globals.maxSize = 6 * 1024^3)
#Load 10x Matrices
ywt.data <- Read10X(data.dir="/wt/outs/filtered_feature_bc_matrix")
fad.data <- Read10X(data.dir="/fad/outs/filtered_feature_bc_matrix")
apc.data <- Read10X(data.dir="/apc/outs/filtered_feature_bc_matrix")
#Create Seurat Objects
ywt <- CreateSeuratObject(counts = ywt.data, project = "ywt", min.cells = 3, min.features = 200)
ywt [["percent.mt"]] <- PercentageFeatureSet(ywt, pattern = "^MT-")
fad <- CreateSeuratObject(counts = fad.data, project = "fad", min.cells = 3, min.features = 200)
fad [["percent.mt"]] <- PercentageFeatureSet(fad, pattern = "^MT-")
apc <- CreateSeuratObject(counts = apc.data, project = "apc", min.cells = 3, min.features = 200)
apc [["percent.mt"]] <- PercentageFeatureSet(apc, pattern = "^MT-")
#Normalize data
ywt <- NormalizeData(ywt, normalization.method = "LogNormalize")
ywt <- FindVariableFeatures(ywt, selection.method = "vst", nfeatures = 2000)
fad <- NormalizeData(fad, normalization.method = "LogNormalize")
fad <- FindVariableFeatures(fad, selection.method = "vst", nfeatures = 2000)
apc <- NormalizeData(apc, normalization.method = "LogNormalize")
apc <- FindVariableFeatures(apc, selection.method = "vst", nfeatures = 2000)
#Scale data
all.genes.ywt <- rownames(ywt)
ywt_scale <-ScaleData(ywt, features = all.genes.ywt)
all.genes.fad <- rownames(fad)
fad_scale <-ScaleData(fad, features = all.genes.fad)
all.genes.apc <- rownames(apc)
apc_scale <-ScaleData(apc, features = all.genes.apc)
#Add metadata
ywt_scale@meta.data[,"treatment"]<- "ywt"
fad_scale@meta.data[,"treatment"]<- "fad"
apc_scale@meta.data[,"treatment"]<- "apc"
#Run PCA
ywt <- RunPCA(ywt_scale, features = VariableFeatures(object = ywt))
fad <- RunPCA(fad_scale, features = VariableFeatures(object = fad))
apc <- RunPCA(apc_scale, features = VariableFeatures(object = apc))
```

```
#Combine Seurat Objects
treatment.raw <- merge(ywt, y = c(fad, apc), add.cell.ids = c("ywt","fad", "apc"),
                        project = "treatment")
treatment.list <-SplitObject(treatment.raw, split.by = "treatment")
reference.list <-treatment.list[c("ywt","fad","apc")]
treatment.anchors <-FindIntegrationAnchors(object.list = reference.list, dims = 1:30)
treatment.integrated <-IntegrateData(anchorset = treatment.anchors, dims = 1:20)
#Set Default assay to integrated
DefaultAssay(treatment.integrated) <- "integrated"
```
